# Enhancing the Antitumor Efficacy of Nisin Through Advanced Nanosystems: A Systematic Review of In Vitro Studies

**DOI:** 10.3390/ph19040611

**Published:** 2026-04-12

**Authors:** Mariatta Ceballos Benavides, Julián Castillo Muñoz, Karol Marcillo Villota, Sinthia Vidal Cañas, Alberto Aragón-Muriel, Jorge A. Egurrola-Pedraza, Yamil Liscano

**Affiliations:** 1 Grupo de Investigación en Salud Integral (GISI), Departamento Facultad de Salud, Universidad Santiago de Cali, Cali 760035, Colombia; mariatta.ceballos00@usc.edu.co (M.C.B.); julian.castillo01@usc.edu.co (J.C.M.); karol.marcillo00@usc.edu.co (K.M.V.); sinthia.vidal00@usc.edu.co (S.V.C.); 2 Grupo de Investigaciones Bioquímicas (GIB), Departamento de Química, Universidad del Magdalena, Santa Marta 470004, Colombia; aaragonm@unimagdalena.edu.co; 3 Grupo de Investigación en Medicina Tropical (CIMET), Facultad de Ciencias de la Salud, Universidad del Magdalena, Santa Marta 470004, Colombia; jegurrola@unimagdalena.edu.co

**Keywords:** nisin, targeted drug delivery, nanosystems, polymeric nanoparticles, cancer therapeutics, formulation strategies, apoptosis, in vitro, systematic review

## Abstract

**Background and Objectives:** While nisin exhibits promising antitumor properties, its clinical utility is hindered by pharmacokinetic instability and rapid enzymatic degradation. This systematic review evaluates the critical role of advanced pharmaceutical formulations and targeted nanosystems in overcoming these limitations to enhance nisin’s cytotoxic and pro-apoptotic efficacy in vitro. **Methods:** Following PRISMA guidelines, a comprehensive search was conducted across six electronic databases (PubMed, ScienceDirect, Scopus, Web of Science, SpringerLink, and DOAJ). In vitro studies comparing free nisin against polymeric, metallic, and cyclodextrin-based nanocarriers across diverse neoplastic lineages were included. Methodological quality was assessed using the SciRAP 2.1 tool, and a within-line comparative analysis was performed for MDA-MB-231 and HT-29 models. **Results:** Twelve studies met the inclusion criteria. A definitive technological inflection point was identified: nisin-loaded nanosystems reduced effective concentrations by up to 2706-fold relative to the free peptide in MDA-MB-231 cells, and 71-fold in A549 lung cancer cells. Mechanistically, nanosystems facilitated membrane pore formation, mitochondrial-mediated apoptosis via Bax/Bcl-2 modulation, caspase 3/7/9 activation, and p53 reactivation. Three previously underreported mechanistic dimensions were identified: TWIST1 downregulation and FZD7 binding in hepatocellular carcinoma, and downregulation of CEA, CEAM6, MMP2F, and MMP9F in colorectal cancer lines. **Conclusions:** The therapeutic viability of nisin in oncology is strictly dependent on pharmaceutical engineering. Future research must prioritize in vivo pharmacokinetic validation, experimental confirmation of novel mechanistic targets, and standardized nisin purity reporting to consolidate its clinical translation.

## 1. Introduction

Cancer comprises a group of diseases characterized by dysregulated cell proliferation, in which genetically transformed cells gain the capacity for local invasion and distant metastasis [1,2,3]. A critical molecular feature distinguishing malignant cells from their healthy counterparts is the aberrant redistribution of phospholipids, particularly the externalization of phosphatidylserine (PS) and phosphatidylethanolamine (PE) on the outer leaflet of the plasma membrane, a phenomenon now recognized as a key determinant of selective cytotoxicity [4,5,6,7]. Projections indicate that the global burden will reach 35 million new cases annually by 2050, representing a 77% increase relative to 2022 levels [8]. This urgency necessitates more effective therapeutic strategies, as conventional chemotherapy and radiotherapy are often limited by low selectivity and systemic toxicity [9,10]. Furthermore, surgical risks and the emergence of drug resistance motivate the investigation of alternative agents with distinct mechanisms of action [11,12].

Bacteriocins, which are ribosomally synthesized antimicrobial polypeptides, have gained attention due to their immunomodulatory potential and favorable safety profiles [11,13,14,15,16]. These agents exploit biochemical differences in membrane charge and lipid composition to achieve selective cytotoxicity [17,18,19,20]. Among studied bacteriocins, nisin, a cationic polycyclic peptide from *Lactococcus lactis*, is the most extensively characterized due to its FDA-approved status as a food preservative and its established toxicological baseline, which facilitates clinical translation [21,22,23]. Naturally occurring variants, such as nisin Z, exhibit enhanced diffusion at neutral pH due to structural substitutions at residue 27, potentially improving efficacy in physiological environments [24,25].

Mechanistically, nisin interacts with anionic phospholipids to form transmembrane pores via the wedge model, leading to cytoplasmic efflux and lactate dehydrogenase (LDH) release [26,27]. Beyond membrane disruption, nisin triggers the intrinsic apoptotic pathway through cytochrome C release and caspase activation [26,28]. However, the clinical translation of free nisin is constrained by pharmacokinetic instability and rapid enzymatic degradation, leading to poor bioavailability at tumor sites [29]. To overcome these barriers, research has focused on advanced delivery nanosystems, including polymeric nanoparticles, gold nanoparticles, and nanosponges, which protect the peptide and enhance cellular uptake [30,31,32]. These platforms represent a qualitative shift in nisin’s antitumor profile, enabling potent effects at substantially lower doses than required for unformulated peptides [33,34,35].

Standardized assays, such as MTT, WST, and LDH, are essential for evaluating metabolic inhibition and membrane damage across experimental models [36]. Apoptosis-specific methods, including Annexin V/PI and morphology staining, further characterize the mechanisms of cell death [25]. This review aims to synthesize in vitro evidence evaluating nisin’s antitumor potential in both free and nano-encapsulated forms. By incorporating a direct within-line comparison of IC_50_ values, this work provides a quantitative framework for evaluating formulation-dependent potency, establishing a foundation for the rational design of future clinical applications.

## 2. Materials and Methods

This systematic review was conducted in strict accordance with the Preferred Reporting Items for Systematic Reviews and Meta-Analyses guidelines [37,38,39]. The methodology was specifically designed to ensure a thorough and unbiased selection of pertinent studies evaluating the antitumor efficacy of nisin in its free form and in advanced nanosystem-based formulations. The research was guided by the following PICO question: In in vitro studies with tumor cell lines (P), does treatment with advanced formulations and nanosystems loaded with nisin (I), compared to free nisin, untreated cells, or cells treated with other agents (C), enhance the reduction in cell viability and the induction of apoptosis (O)? This review has been prospectively registered in the International Prospective Register of Systematic Reviews under the registration number CRD420261320553 (See Supplementary Table S1: PRISMA Checklist).

### 2.1. Eligibility Criteria

The selection of studies followed strict inclusion and exclusion parameters to ensure experimental reliability. Specifically, studies were required to include valid control groups and complete data sets for cell viability or apoptosis markers to be considered for synthesis. The detailed criteria are presented in Table 1.

### 2.2. Information Sources and Search Strategy

An exhaustive and systematic search was performed across six major electronic databases to identify all potentially relevant studies. The databases consulted were PubMed, ScienceDirect, Scopus, Web of Science, SpringerLink, and the Directory of Open Access Journals (DOAJ)**.** In addition to database searches, the reference lists of all retrieved articles that met the inclusion criteria were manually reviewed to identify studies that might have been overlooked by the algorithmic search strategy.

### 2.3. Search Strategy

A comprehensive search strategy was developed by combining controlled vocabulary terms, including Medical Subject Headings (MeSH) where applicable, with free-text terms, applying Boolean operators (AND, OR) and filters for study type. As an illustrative example, the search string employed in PubMed was as follows: (“nisin” OR “nisina”) AND (“cancer” OR “neoplasm” OR “tumor”) AND (“apoptosis” OR “cell proliferation” OR “Bax/Bcl-2” OR “caspase”) AND (“in vitro” OR “cell line”). This search algorithm was systematically adapted to the specific syntax and indexing conventions of each database. All searches were conducted in November 2025, and the complete search strings for each database are available upon request. The Rayyan Intelligent Systematic Review platform (https://www.rayyan.ai/, accessed on 15 November 2025) 2025 version was used for the storage, deduplication, and collaborative management of retrieved records.

### 2.4. Study Selection

**Figure 1:**
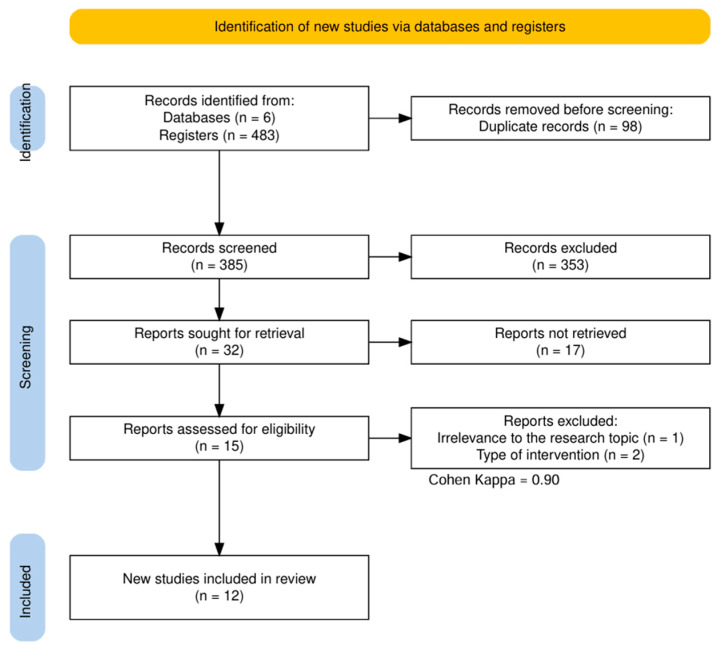
PRISMA flow diagram. The study selection process involved the screening of 483 records, with an inter-rater agreement measured by a Cohen’s kappa coefficient of 0.90, which indicates excellent consensus among the reviewers.

The study selection process was conducted in two sequential phases. In the first phase, three reviewers, M.C.B., J.C.M., and K.M.V., independently evaluated the titles and abstracts of all identified records to determine their eligibility against the predefined criteria. In the second phase, full-text reports of all studies that appeared to meet the inclusion criteria, or for which eligibility remained unclear from the abstract alone, were retrieved and subjected to independent full-text review by the same three reviewers. Discrepancies arising between reviewers at either phase were resolved through structured discussion, and a fourth reviewer, Y.L., was consulted when consensus could not be reached through discussion alone. To quantify the degree of inter-reviewer agreement in study selection and assess the reliability of the screening process, Cohen’s kappa coefficient was calculated, with a value of κ = 0.90 indicating excellent agreement among reviewers, as reported in the PRISMA flow diagram (Figure 1).

### 2.5. Data Extraction

A standardized data extraction form was designed prior to the screening process to ensure consistency, completeness, and reproducibility in data collection across all included studies. Three reviewers, M.C.B., J.C.M., and K.M.V., independently extracted the following categories of information from each eligible study: study characteristics, comprising the first author, year of publication, country of origin, and overall experimental design; neoplasm characteristics, including tumor type, cell line identity, and authentication or validation method; intervention details, with specific attention to pharmaceutical formulation type, nanocarrier composition, nisin variant and form, administered dose range, exposure duration, control groups employed, and number of experimental replicates; primary outcomes, focusing on quantitative measures of cell viability reduction and apoptosis induction; results, encompassing key findings, statistical significance thresholds, and authors’ conclusions; and reported limitations. Upon completion of independent extraction by the three primary reviewers, a fourth reviewer, Y.L., verified the integrity, accuracy, and internal consistency of all recorded data, resolving any remaining discrepancies. The PRISMA 2020 flow diagram presented in Figure 1 was generated using the PRISMA2020 R package 4.3.3 [40], accessed on 15 November 2025.

### 2.6. Risk of Bias and Methodological Quality Assessment

The risk of bias assessment for all included studies was performed independently by three reviewers, M.C.B., J.C.M., and K.M.V., using the SciRAP 2.1 tool, which is a validated instrument specifically designed for the evaluation of reliability and relevance in in vitro toxicological studies [41]. The SciRAP tool was selected over alternative quality assessment instruments because of its domain-specific applicability to cell-based experimental designs, which constitute the entirety of the evidence base synthesized in this review. Three principal domains were evaluated for each study: reporting quality, methodological quality, and experimental relevance. Within each domain, individual criteria were rated as fulfilled, partially fulfilled, or not reported, in accordance with the predetermined operational guidelines of the SciRAP framework. Discrepancies between reviewers were addressed through collaborative discussion, with recourse to Y.L. as a final arbiter when agreement was not achieved through discussion.

### 2.7. Data Synthesis

Given the considerable heterogeneity among the included studies, encompassing diverse neoplastic cell lines, a wide range of nisin concentrations and formulation types, variable exposure durations, and different cytotoxicity and apoptosis quantification methods, a quantitative meta-analysis was not considered methodologically appropriate. Accordingly, a qualitative synthesis in narrative format was selected as the primary analytical approach, supplemented by structured summary tables presenting the key characteristics, interventions, outcomes, and quality assessments of the included studies. To ensure the technical comparability of results across investigations employing identical cell lines, a dedicated within-line comparative analysis was incorporated into the findings, focusing on the studies utilizing HT-29 and MDA-MB-231 cells, with particular emphasis on the consistency and formulation-dependence of the standardized IC_50_ values.

### 2.8. Ethical Considerations

As this is a review of published in vitro studies, approval from an ethics committee was not required. This review did not involve experiments with humans or animals.

## 3. Results

### 3.1. Characteristics of the Included Studies

A total of 483 records were identified across six databases, with 98 duplicates removed, leaving 385 records. Of these, 353 were excluded based on title and abstract screening, utilizing a peer-review process with a Cohen’s kappa coefficient of 0.90, which indicates excellent agreement among reviewers. Subsequently, 32 records were evaluated for eligibility through full-text reviews. Among these, one record was excluded for not meeting relevance criteria, two records due to the required study type, and 17 due to insufficient data. As a result, 12 articles were finally included in this systematic review, as shown in Figure 1.

### 3.2. Overview of Study Findings

The 12 included studies were conducted across diverse geographical regions, including India, Iran, South Africa, Turkey, and South Korea, utilizing various cell lines to investigate the mechanisms of action of nisin. The primary focus of these investigations was the evaluation of cell viability and apoptosis induction across different neoplasms. The consistent application of standardized criteria in the selection of cell lines allowed for a robust comparison between studies, minimizing variability in cell populations. Notably, eight of the 12 studies provided high-resolution data on the MDA-MB-231 (breast cancer) and HT-29 (colon cancer) lines, which served as the primary models for within-line comparative analysis. Furthermore, to address the methodological heterogeneity identified during the review, all cytotoxic values were standardized to μg/mL, facilitating a more reliable and clearer understanding of the mechanisms of action and potency of nisin-loaded nanosystems in oncological contexts.

### 3.3. Characteristics of the Studied Neoplasms

The experimental models utilized across the 12 included studies encompass a diverse range of neoplastic lineages, representing a broad spectrum of human malignancies. As detailed in Table 2, the research targeted various tumor types, including colorectal, breast, lung, glioblastoma, hepatocarcinoma, melanoma, prostate, and pancreatic cancers. The specific cell lines employed were HT-29, MCF-7, SF-767, SNU182, HuH-7, MDA-MB-231, A375, A549, HCT116, LS180, SW48, H1299, SW-620, and PC-3, among others. These models exhibit significant genetic and metabolic heterogeneity, which allows for a comprehensive evaluation of nisin’s broad-spectrum capacity to reduce cell viability and promote programmed cell death. Notably, colorectal and breast cancer models were the most frequently investigated, appearing in approximately 42% and 58% of the reviewed literature, respectively, providing a concentrated baseline for comparing the relative potency of free nisin against advanced pharmaceutical formulations.

### 3.4. Intervention and Control Details

The experimental interventions across the 12 reviewed studies involved a diverse array of nisin concentrations and formulations, engineered to evaluate antitumor efficacy across distinct neoplastic models. These interventions varied significantly in terms of nisin variants (A, Z, or ZP), chemical modifications, such as glycation, and the utilization of ligand-mediated or responsive nanocarriers. Such factors, alongside exposure duration and the choice of comparative controls, fundamentally dictate the observed cytotoxic profiles and allow for a comprehensive synthesis of nisin’s therapeutic range, as detailed in Table 3.

Investigations conducted by Ahsan et al., 2024 [48], Balcik-Ercin & Sever, 2022 [49], Norouzi et al., 2018 [44], and Mohan Latha Kumari et al., 2025 [45] utilized free nisin as the primary intervention to establish baseline dose-dependent effects, with Lewies et al., 2018 [27] specifically employing high-purity nisin Z (95%) to minimize confounding factors from impurities. Furthermore, Patil & Kunda, 2022 [47] explored the efficacy of the nisin ZP variant in lung cancer models at concentrations up to 250 μM. A significant chemical advancement was identified in the study by Mohammadi et al., 2024 [42], which demonstrated that glycation of nisin A significantly enhances the peptide’s cytotoxic potency and stability, particularly during extended exposure periods of up to 72 h.

The application of advanced nanosystems focused on overcoming pharmacokinetic barriers and achieving targeted delivery. Haider et al., 2020 [34] utilized SPN-NPs to exploit the high affinity of spectrin for tumor-specific membrane markers, while Haider et al., 2022 [46] employed nisin-PLGA nanoparticles (NPN) to broaden the therapeutic window against multiple tumor lines. Targeting precision was further refined by Saravanakumar et al., 2024 [35] through the development of glutathione-responsive gold nanoparticles, which release nisin specifically within the reductive tumor microenvironment. Additionally, Khazaei Monfared et al., 2022 [25] successfully stabilized nisin Z within β-cyclodextrin nanosponges cross-linked with PMDA and CDI, and Salehi et al., 2024 [43] achieved a synergistic antiproliferative effect by co-encapsulating nisin and curcumin within polymersomes prepared via microfluidic technology.

Intervention durations typically ranged from 24 to 48 h, although extended kinetics up to 72 h were observed in studies focusing on glycated formulations. All research designs incorporated negative controls, frequently employing non-malignant cell lines, such as CHO, HaCaT, HDF, fR2, and NIH3T3, to assess selectivity. Positive controls utilized conventional chemotherapeutics, including 5-fluorouracil, oxaliplatin, doxorubicin, and paclitaxel, or membrane-disrupting agents, such as Triton-X. This methodological diversity underscores the current shift toward pharmaceutical engineering, where the integration of nisin into specialized nanocarriers consistently improves its stability and clinical potential.

### 3.5. Cell Viability Results

The reviewed studies evaluated cell viability using diverse biochemical assays, including MTT, LDH, SRB, and Trypan Blue (TR), as summarized in Table 4. To ensure a rigorous comparative analysis across the 12 included studies, this review focuses on the standardized IC_50_ value. This parameter is critical for determining the biological effectiveness of a compound, as it enables the comparison of potency between different agents or formulations under standardized conditions Srinivasan & Lloyd, 2024 [36]. To address the unit heterogeneity identified in the original literature, all reported molar and international unit (IU) values were converted to μg/mL using the molecular weight of nisin (3354.07 g/mol), providing a unified quantitative framework for this systematic synthesis.

Investigating the influence of pharmaceutical engineering on potency, Khazaei Monfared et al., 2022 [25] utilized MTT and LDH assays to demonstrate that nisin-loaded nanosponges cross-linked with PMDA exhibit significantly higher cytotoxicity against HT-29 and MCF-7 cell lines compared to free nisin Z. This effect was particularly pronounced in HT-29 cells, where viability was reduced to approximately 20% at 250 μg/mL after 24 h. Similarly, Lewies et al., 2018 [27] reported a dose-dependent decrease in A375 melanoma cell viability, identifying an IC_50_ of 188.5 ± 8.7 μM (standardized to 632.24 μg/mL). Notably, this study confirmed a higher IC_50_ of 439 ± 8.3 μM for non-malignant keratinocytes (HaCaT), establishing a clear therapeutic window for nisin in skin cancer models.

#### Comparative Analysis by Cell Line

To facilitate a rigorous assessment of nisin’s effectiveness, findings were grouped by identical cell lines, with a primary focus on MDA-MB-231 and HT-29, which were the most frequently evaluated models (See Table 5).

Breast Cancer Models (MDA-MB-231 and MCF-7)

For the MDA-MB-231 triple-negative breast cancer (TNBC) line, four independent studies demonstrated a massive potency increase through formulation. Haider et al. 2020 [34] recorded an IC_50_ of 162.38 μg/mL for free nisin, which was reduced to 0.06 μg/mL using PLGA-SPN NPs, representing a fold-reduction of >2706×. This formulation achieved a high Selectivity Index (SI > 2383×) by targeting externalized PS/PE on the tumor membrane. Mohammadi et al. 2024 [42] corroborated this susceptibility, reporting that glycated nisin achieved an IC_50_ of 0.05 μg/mL at 72 h, an approximately 233-fold improvement over the free peptide, inducing apoptosis in 73–81% of cells. In contrast, unconjugated PLGA nanoparticles Haider et al., 2022 [46] showed a more moderate 12.5× improvement (IC_50_ = 13.0 μg/mL), while glutathione-responsive gold nanoparticles Saravanakumar et al., 2024 [35] exhibited lower sensitivity in this specific line compared to lung models.

In MCF-7 (Luminal) cells, Salehi et al. 2024 [43] identified a synergistic effect (CI < 1) by co-delivering nisin and curcumin in polymersomes, reducing the LD_50_ from 43.56 μg/mL to 18.20 μg/mL. Khazaei Monfared et al. 2022 [25] noted that while β-cyclodextrin nanosponges (PMDA-crosslinked) significantly reduced viability, the cellular uptake in MCF-7 was lower compared to colorectal models.

Colorectal Adenocarcinoma (HT-29 and LS180)

The HT-29 line served as a critical model for identifying novel molecular targets. Mohan Latha Kumari et al. 2025 [45] reported the first instance of p53 reactivation by nisin (IC_50_ = 68.09 μg/mL), which triggered G0/G1 arrest and caspase 3/7/9 activation. Norouzi et al. 2018 [44] highlighted the anti-metastatic potential of free nisin in HT-29 and LS180 lines by demonstrating the downregulation of CEA, CEAM6, and MMP genes. Furthermore, Khazaei Monfared et al. 2022 [25] found that HT-29 cells were more susceptible to β-cyclodextrin nanosponges than breast cancer lines, showing intense LDH release and late-stage apoptosis.

Lung Cancer and Other Neoplasms

In A549 lung cancer cells, Saravanakumar et al. 2024 [35] achieved a 71-fold potency increase using GSH-responsive gold nanoparticles (IC_50_ = 0.88 μg/mL vs. 62.5 μg/mL for free nisin). Patil & Kunda 2022 [47] confirmed that nisin-ZP induces p53-independent cell death in both A549 and H1299 (p53-null) lines, effectively inhibiting 3D tumor spheroids.

For other neoplasms, nisin demonstrated the following:

- Glioblastoma (SF-767): Selective inhibition (IC_50_ = 30.65 μg/mL) with a selectivity index of 3.6× versus CHO cells [48].
- Hepatocellular Carcinoma (HuH-7/SNU182): Significant inhibition of the mesenchymal phenotype through TWIST1 downregulation and FZD7 protein interaction [49].
- Melanoma (A375): Bioenergetic collapse and ROS generation, with a 2.3× selective window over healthy HaCaT keratinocytes [27].

### 3.6. Apoptosis Rate Results

The induction of programmed cell death was evidenced through various specialized techniques, including Annexin V/PI flow cytometry, AO/EB staining, ELISA, DAPI nuclear staining, and Western blot analysis. While some studies, specifically those by Ahsan et al., 2024 [48], Haider et al., 2022 [46], and Norouzi et al., 2018 [44], focused primarily on metabolic viability, the remaining literature provided a detailed narrative of nisin’s pro-apoptotic mechanisms. A critical finding across these studies is that the transition from free nisin to nisin-loaded nanosystems significantly amplifies the apoptotic index, shifting the cellular response from simple membrane lysis toward regulated cell death pathways.

In breast cancer models, particularly the MDA-MB-231 line, Mohammadi et al., 2024, demonstrated that glycated nisin achieves a superior apoptosis rate compared to the free peptide, with doses of 10 to 40 μg/mL yielding an apoptotic population of 73% to 81% after only 24 h [42]. This intensified response is further elucidated by the work of Haider et al., 2020 [34], who reported that targeted PLGA nanoparticles induce apoptosis via the activation of the Bak/Bax signaling pathway and a concomitant down-regulation of the anti-apoptotic protein Bcl-2. Similarly, Salehi et al., 2024 [43] observed that the co-delivery of nisin and curcumin within polymersomes resulted in a higher apoptosis rate in MCF-7 cells than free nisin treatments, suggesting that the synergistic interaction and the controlled release kinetics of the nanocarrier are fundamental to overcoming cellular resistance.

Mechanistic depth was notably enhanced in colorectal cancer studies, where Mohan Latha Kumari et al., 2025 [45] provided the first evidence of nisin-mediated reactivation of mutated p53 in HT-29 cells, accompanied by the activation of executioner caspases 3 and 7 and the initiator caspase 9. These results complement the findings of Khazaei Monfared et al., 2022 [25], who utilized flow cytometry to distinguish between the responses of different lineages, noting that while HT-29 cells predominantly undergo late apoptosis or necrosis, MCF-7 cells exhibit markers of early apoptotic activation when treated with nisin Z-loaded nanosponges. The superiority of PMDA-cross-linked nanosponges in these models highlights the “technological inflection point” where pharmaceutical modification directly dictates the precision of the antitumor mechanism.

Apoptosis induction was also confirmed in lung, liver, and melanoma models. Patil & Kunda, 2022 [47] found that nisin ZP treatment in A549 and H1299 lung cancer cells produced an apoptotic population of approximately 45% after 48 h. In the same lineage, Saravanakumar et al., 2024 [35] reported that glutathione-responsive gold nanoparticles modified with cysteamine significantly enhanced chromatin condensation and membrane blebbing. For hepatocarcinoma lines, Balcik-Ercin & Sever, 2022 [49] evidenced significant apoptotic shifts, particularly in HuH-7 cells (44.2% at 320 μg/mL), while Lewies et al., 2018 [27] described a dual mechanism in A375 melanoma cells involving mitochondrial bioenergetic failure and ROS-mediated late apoptosis at concentrations exceeding 100 μM. Collectively, these data reinforce the conclusion that nisin, particularly when integrated into advanced delivery platforms, acts as a multi-target apoptotic enhancer across a wide array of human malignancies.

### 3.7. Risk of Bias and Methodological Quality Results

**Figure 2:**
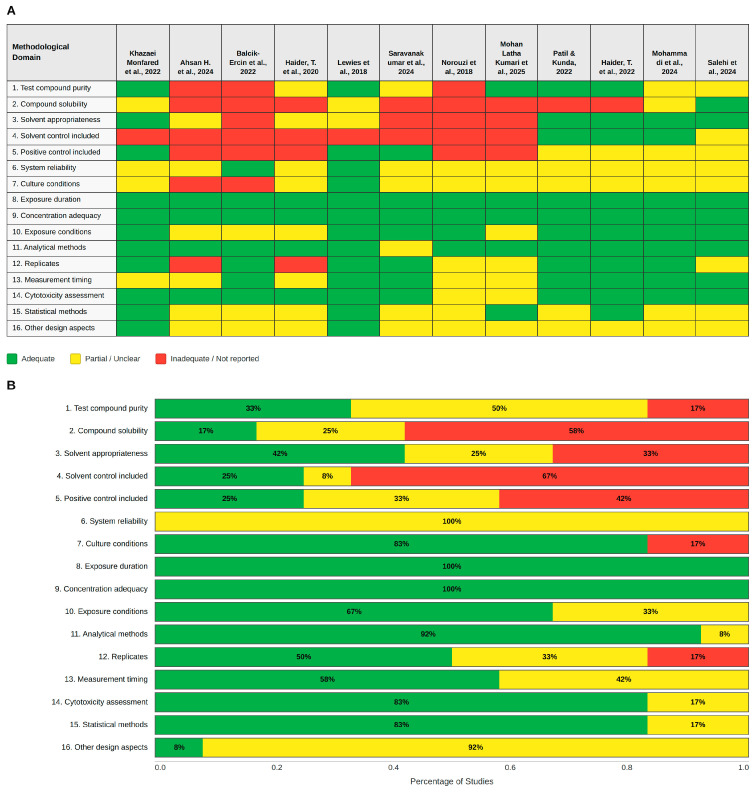
Methodological quality and risk of bias assessment. (**A**) Risk of bias summary: Review of authors’ judgments on key methodological aspects for each study based on the SciRAP 2.1 tool. Green indicates total fulfillment, yellow represents partial fulfillment with deficiencies, and red indicates non-fulfillment or lack of reporting. (**B**) Risk of bias graph: Summary of the percentage of studies categorized as fulfilled, partially fulfilled, or not fulfilled for each methodological criterion. Figure created using R software version 4.3.3 (accessed on 15 November 2025, https://cran.r-project.org/bin/windows/base/) [25,27,34,35,42,43,44,45,46,47,48,49].

The risk of bias assessment, conducted using the SciRAP 2.1 tool for in vitro studies, revealed significant methodological variability across the 12 included reports. While the global quality assessment indicates that many studies align with fundamental scientific standards, specific domains related to reporting transparency and experimental rigor highlight critical areas for improvement in future research. As illustrated in Figure 2B, the primary strengths of the included studies lie in their experimental design and outcome measurement, where 100% of the studies fulfilled the criteria for exposure duration and concentration adequacy, providing a robust baseline for evaluating dose–response relationships. Analytical rigor was also notable, with analytical methods reaching 92% fulfillment, while cytotoxicity assessment and statistical methods both achieved 83%, ensuring that the primary antitumor effects were analyzed with adequate technical precision.

Conversely, significant gaps were identified in the reporting of foundational chemical and experimental control domains, particularly regarding the inclusion of solvent controls, which represented the most significant weakness since 67% of the studies failed to report or include them. Furthermore, positive controls were not fulfilled in 42% of the cases, and compound solubility was not adequately addressed in 58% of the studies. Compound characterization also showed room for improvement, as test compound purity was fully documented in only 33% of the reports, with 50% providing only partial information. As observed in the summary heatmap in Figure 2A, studies such as Balcik-Ercin et al. (2022) [49] and Norouzi et al. (2018) [44] exhibited a higher density of non-fulfillment in these critical domains compared to the more robust methodological profiles found in Patil and Kunda (2022) [47] or Mohammadi et al. (2024) [42]. System reliability was categorized as partially fulfilled across 100% of the studies, suggesting that while experimental setups were functional, detailed validation of the biological system stability was often underspecified. These findings underscore the urgent need for more transparent reporting in nisin-related in vitro research to ensure the reproducibility of results and facilitate the transition from laboratory evidence toward clinical applications.

## 4. Discussion

### 4.1. Main Findings

This systematic review synthesizes twelve independent in vitro investigations evaluating the antitumor effects of nisin across eight neoplastic lineages, with a particular emphasis on how pharmaceutical engineering modulates cytotoxic potency and apoptotic induction across multiple cancer models. Although a dose-dependent reduction in cell viability was consistently observed across all included studies, the inherent heterogeneity of experimental parameters, including detection methodologies, nisin preparation purity, and dosage intervals, complicates the definition of a universal therapeutic concentration for the unformulated peptide. Nevertheless, the evidence converges on a definitive technological inflection point: the transition from free nisin to advanced nanosystems represents a qualitative, not merely quantitative, shift in antineoplastic efficacy.

The most compelling demonstration of this inflection is provided by Haider et al. 2020 [34], whose PLGA-SPN NPs reduced the IC_50_ against MDA-MB-231 cells from 162.38 µg/mL to 0.06 µg/mL, a reduction exceeding 2706×-fold, while simultaneously achieving a selectivity index greater than 2383× relative to normal breast epithelial cells (FR-2). This result demonstrates that active targeting of tumor-specific membrane markers, specifically externalized PS and PE, is not merely an incremental improvement but a transformative therapeutic strategy. Comparable evidence was provided by Saravanakumar et al. 2024 [35], whose glutathione-responsive gold nanoparticles achieved an IC_50_ of 0.88 µg/mL against A549 lung cancer cells, a 71-fold reduction relative to free nisin (62.5 µg/mL), through a tumor microenvironment-triggered release mechanism that exploits the elevated intracellular GSH concentrations characteristic of cancer cells. Mohammadi et al. 2024 [42] further demonstrated that even chemical modification without nanoencapsulation, specifically glycation of nisin A, achieves an approximately 233-fold potency increase at 72 h (IC_50_ = 0.05 µg/mL vs. 11.64 µg/mL for the free peptide), with 73 to 81% of MDA-MB-231 cells entering apoptosis at doses of 10 to 40 µg/mL.

Beyond potency, this review identifies three mechanistic dimensions previously underrepresented in the nisin literature. First, Mohan Latha Kumari et al. 2025 [45] reported the first evidence of p53 reactivation by nisin in HT-29 colorectal cells, accompanied by G0/G1 cell cycle arrest and sequential caspase 3/7/9 activation. Second, Balcik-Ercin and Sever 2022 [49] demonstrated that nisin suppresses TWIST1 expression in hepatocellular carcinoma cells, implicating epithelial–mesenchymal transition (EMT) inhibition as a resistance-reversal mechanism, and provided the first molecular docking evidence of nisin A binding to FZD7 (Wnt pathway receptor, docking score −6.23 kcal/mol), a target overexpressed in primary hepatocellular carcinoma, where its upregulation promotes proliferation, invasion, and drug resistance through activation of the Wnt/β-catenin signaling pathway [50]. Third, Norouzi et al. (2018) [44] characterized nisin as an anti-metastatic agent by documenting significant downregulation of CEA, CEAM6, MMP2F, and MMP9F at both gene (qRT-PCR) and protein (ELISA) levels in colorectal cancer lines, extending its therapeutic relevance well beyond direct cytotoxicity, consistent with findings from independent anti-adhesion studies [51].

### 4.2. Comparison with Previous Studies

The antitumor potential of bacteriocins has been documented across multiple neoplastic lineages, yet the present review reinforces that pharmaceutical formulation, and not intrinsic peptide potency, is the decisive factor for clinical viability. Other bacteriocins, such as Plantaricin BM-1 and Colicin N, have demonstrated pro-apoptotic activity in colorectal and lung cancer models, respectively [21,52,53,54,55]; however, when compared with the nanosystem data synthesized here, properly formulated nisin consistently achieves equivalent or superior efficacy at substantially lower concentrations. For instance, whereas free nisin may require concentrations approaching 4000 µg/mL to induce a significant Bax/Bcl-2 shift in certain colorectal models, the PLGA-SPN system of Haider et al. 2020 [34] achieves the same molecular trigger at 0.06 µg/mL, a difference of approximately five orders of magnitude.

The within-line comparative analysis performed in this review, enabled by four independent MDA-MB-231 datasets and four independent HT-29 datasets, reveals that potency variation across studies employing the same cell line largely reflects methodological factors, including assay type, nisin purity, and exposure duration, rather than genuine biological inconsistency. This finding has direct implications for study design: the continued use of the same commercial nisin preparation across studies is not equivalent to the use of the same compound if lot-specific purity is not documented, as illustrated by the non-convertible IU/mL values reported by Norouzi et al. 2018 [44]. This review, therefore, distinguishes itself from previous qualitative syntheses by providing an audited, within-line dataset that explicitly separates formulation effects from methodological artifacts, establishing a more reliable baseline for future comparative pharmacokinetic studies. Additionally, the selective efficacy of formulated nisin provides a biocompatible biological alternative to synthetic compounds such as pyrimidodiazepines [56], targeting similar neoplastic vulnerabilities through a nanotechnological framework that preserves the GRAS status of the original peptide.

Two primary publications warrant a rigorous comparative analysis with the present work. Hosseini et al. 2022 [57] conducted a systematic review focused exclusively on the cytotoxicity of nisin in its free peptide form. Their analysis of fifteen studies published between 2003 and 2020 identified differential cytotoxic activity across various cancer lines, reporting the highest efficacy against A431, A375, and LS180. The present review advances this foundational work in several critical dimensions: it extends the search horizon to 2025, capturing contemporary nanosystem-based evidence unavailable during the 2020 search cutoff, it utilizes the SciRAP 2.1 tool for formal methodological quality assessment to provide an auditable layer of transparency absent from earlier syntheses, it establishes a within-line comparative framework for MDA-MB-231 and HT-29 that quantifies formulation-dependent potency differences of up to 2706-fold, which is a magnitude previously unreported in nisin literature, and it identifies specific mechanistic dimensions, including p53 reactivation, TWIST1/EMT suppression, and FZD7 molecular docking, that were either unpublished or not captured by prior search parameters.

A second point of comparison is the review by Zhang et al. 2024 [58], which focuses on albumin-based nanosystems designed to exploit the tumor microenvironment. While that work provides a comprehensive architectural framework for albumin carrier design, its primary focus is the engineering of nanocarriers for conventional therapeutic agents, such as chemotherapy, phototherapy, and radiotherapy, rather than evaluating the intrinsic antineoplastic activity of the cargo molecule itself. Nisin occupies a distinct position within this landscape because, unlike passive chemotherapeutic cargos, it possesses independent membrane-disruptive and pro-apoptotic activity while simultaneously benefiting from nanosystem-mediated protection and targeted delivery. Furthermore, nisin’s FDA-recognized GRAS status as a non-toxic food preservative provides a pre-established toxicological baseline that conventional synthetic chemotherapeutics lack, which may significantly shorten regulatory timelines for the clinical translation of nisin-loaded formulations.

### 4.3. Mechanisms of Action of Nisin

**Figure 3:**
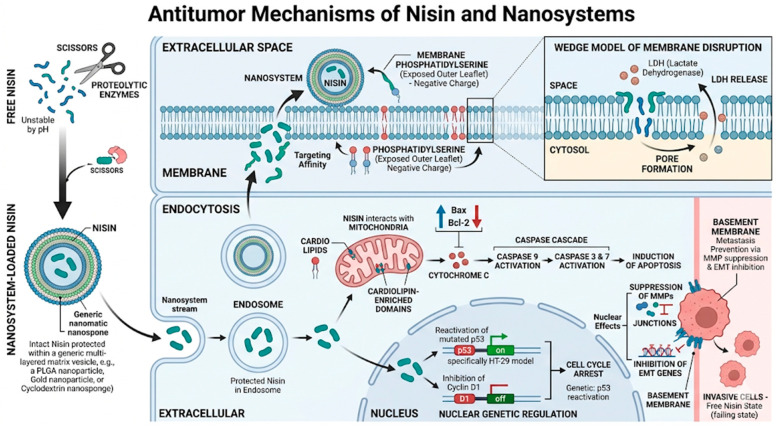
Comparative antitumor mechanisms of free and nanoformulated nisin. The diagram illustrates how nanosystems protect nisin from proteolytic degradation, enabling targeted delivery. Key mechanisms include membrane pore formation via the wedge model (LDH release) and mitochondrial-mediated apoptosis involving cytochrome C release, caspase activation (9, 3, and 7), and Bax/Bcl−2 modulation. Intracellularly, nisin-loaded systems facilitate p53 reactivation and cyclin D1 inhibition, leading to cell cycle arrest. Additionally, the suppression of MMPs and EMT genes effectively inhibits metastatic progression and basement membrane invasion. Source: own elaboration with NanoBanana version Pro. Arrow legend: Solid straight arrows (→) indicate the direction of biological processes or signaling pathways; green upward arrows (↑) denote upregulation or overexpression; red downward arrows (↓) denote downregulation or suppression; curved arrows represent sequential multistep cascading events. Source: own elaboration with NanoBanana Pro (https://nanobanana.co/, accessed on 5 April 2026).

The primary mechanism of action involves nisin’s selective affinity for the anionic outer leaflet of neoplastic cell membranes, where externalized phosphatidylserine, present at three to seven times higher concentrations in tumor cells than in normal cells, serves as the principal docking site [11,12,59,60,61]. Membrane binding initiates transmembrane pore formation through the wedge model of membrane disruption, producing rapid efflux of LDH, as directly corroborated by Khazaei Monfared et al. 2022 [25] and Lewies et al. 2018 [27]. As illustrated in Figure 3, advanced pharmaceutical formulations are critical at this initial phase: nanoencapsulation shields the cationic peptide from proteolytic degradation and pH-induced inactivation in biological fluids, ensuring that intact nisin reaches the tumor cell surface at sufficient local concentrations to initiate pore formation without premature neutralization.

Intracellularly, nisin targets cardiolipin-enriched domains of the mitochondrial outer membrane, triggering mitochondrial outer membrane permeabilization (MOMP) and the release of cytochrome C into the cytosol [62,63]. This activates the canonical intrinsic apoptosis cascade: cytochrome C assembles the apoptosome, which recruits and cleaves procaspase 9 (initiator), which in turn activates executioner caspases 3 and 7, all three confirmed by ELISA in HT-29 cells by Mohan Latha Kumari et al. 2025 [45]. Concomitantly, nisin shifts the Bax/Bcl-2 ratio in favor of apoptosis, as demonstrated by Western blot in MDA-MB-231 cells treated with PLGA nanoparticles by Haider et al. 2022 [46]. Nanosystems amplify this mitochondrial response by facilitating cellular uptake via endocytosis, allowing the peptide to bypass initial membrane barriers and achieve sustained intracellular release, as evidenced by the significantly elevated intracellular ROS production and DΨm depletion observed in nanoparticle-treated cells relative to free nisin across three independent studies [46,64,65,66].

Beyond direct cytotoxicity, nisin participates in complex nuclear and genetic regulation. As shown in Figure 3, nisin mediates the reactivation of mutated p53 in colorectal models such as HT-29 [45,51], while simultaneously inhibiting cyclin D1 to promote G0/G1 cell cycle arrest. At the invasive level, nisin suppresses the expression of CEA, CEAM6, MMP2F, and MMP9F [44,67], genes that govern basement membrane degradation and metastatic dissemination, and inhibit TWIST1-mediated EMT in hepatocellular carcinoma [49]. The first computational evidence of nisin A binding to FZD7, a Wnt signaling receptor overexpressed in primary hepatocellular carcinoma, was provided by Balcik-Ercin and Sever 2022 [49], opening a novel mechanistic axis that warrants experimental validation. Furthermore, Lewies et al. 2018 [27] provided the most complete bioenergetic characterization of nisin’s effects using the Seahorse XF Analyzer, demonstrating that nisin-Z causes dose-dependent collapse of basal respiration, maximal respiratory capacity, ATP production, and spare respiratory capacity in A375 melanoma cells, with compensatory glycolysis upregulated at low doses (50 µM) and glycolytic capacity also suppressed at higher doses (≥150 µM), constituting a profound metabolic shutdown that precedes cell death.

### 4.4. Limitations of the Included Studies

The analyzed studies present several limitations that must be acknowledged to contextualize the evidence and guide future research. Most critically, none of the 12 included studies provided in vivo validation, which restricts the assessment of nanotechnological advantages in complex biological environments where pharmacokinetic barriers, immune interactions, and tumor microenvironment dynamics substantially alter peptide behavior [68,69]. Methodologically, 67% of studies failed to report or include solvent controls, 58% did not adequately characterize compound solubility, and 50% only partially documented test compound purity, all domains identified as insufficient by the SciRAP 2.1 assessment. These gaps are particularly consequential for nisin studies because commercial preparations, typically 2.5% *w*/*w*, contain substantial impurities that confound dose calculations and prevent unit standardization, as exemplified by the IU/mL values from Norouzi et al. 2018 [44] that cannot be converted to µg/mL without lot-specific purity data. Additionally, the absence of positive controls in 42% of studies limits the ability to contextualize nisin’s potency relative to established chemotherapeutic benchmarks. The restriction of all studies to 2D monolayer models under standard culture conditions, with only Patil and Kunda 2022 [47] including 3D spheroid data, represents a significant translational gap that future research must address.

Furthermore, the absence of standardized reporting for nisin variants (nisin A vs. nisin Z) across the included studies represents an additional limitation, as structural differences between variants may differentially influence membrane affinity and cytotoxic potency.

### 4.5. Limitations of the Review

This review is limited by the exclusion of unpublished literature and studies in languages other than English or Spanish, which may introduce publication bias toward positive results. The extreme heterogeneity in nanosystem architectures, ranging from polymeric PLGA nanoparticles and β-cyclodextrin nanosponges to gold nanoparticles and polymersomes, precluded a quantitative meta-analysis, as the mechanistic and physicochemical differences between systems prevent meaningful pooling of IC_50_ values across formulations. Furthermore, the use of different cytotoxicity assays (MTT, SRB, WST, Alamar blue, Trypan blue) across studies introduces systematic measurement differences that cannot be fully resolved by unit standardization alone. Two methodological corrections applied in this version of the manuscript must be noted explicitly. The IC_50_ values from Norouzi et al. 2018 [44] were originally reported in IU/mL, and a previously circulating conversion to µg/mL (1.15 to 5.75 µg/mL) has been removed from this version, as such conversion is not feasible without lot-specific nisin purity data. Additionally, the cytotoxicity metric reported by Salehi et al. 2024 [43] represents LD_50_ values calculated using CompuSyn software (ComboSyn, Inc., Paramus, NJ, USA; https://www.combosyn.com, accessed on 5 April 2026) (Chou-Talalay method), not IC_50_, and should not be compared directly with IC_50_ values from other studies; the normal cell line in that study has also been corrected from HFF to HDF (Human Dermal Fibroblasts) in accordance with the original publication. Despite these constraints, the rigorous within-line comparative framework applied here and the systematic application of the SciRAP 2.1 tool across all 12 studies provide a more transparent and auditable qualitative synthesis than previously available. Additionally, gray literature, conference proceedings, and preprint repositories were not systematically searched, which may further contribute to publication bias.

### 4.6. Clinical Implications

The findings of this review indicate that the clinical future of nisin as an antineoplastic agent is inextricably linked to pharmaceutical technology. Free nisin, in its current commercial form, exhibits insufficient potency and stability for systemic oncological application: concentrations required to achieve 50% inhibition in most cancer lines, ranging from 100 to 500 µg/mL, would necessitate systemic doses that are pharmacokinetically implausible and potentially toxic to normal tissues. By contrast, nanosystem-formulated nisin consistently achieves therapeutic IC_50_ values below 15 µg/mL, and in the case of SPN-NPs, below 0.1 µg/mL, while maintaining high selectivity for cancer versus normal cells. This selectivity is mechanistically grounded in the asymmetric phospholipid distribution of cancer cell membranes, a vulnerability that is actively exploited by PS/PE-targeting ligands and passively by GSH-responsive release systems.

Beyond cytotoxicity, the anti-metastatic and anti-EMT activities documented for nisin [44,49] suggest that nisin-based formulations may have particular utility in resistant, invasive cancer phenotypes, precisely the contexts where conventional chemotherapy is least effective. Triple-negative breast cancer (MDA-MB-231) and colorectal adenocarcinoma (HT-29) [70], the two best-characterized models in this review, represent high-unmet-need indications where the biocompatibility and GRAS status of nisin are additional clinical advantages over synthetic cytotoxic agents. The documented synergistic effect of nisin combined with curcumin in polymersomes further suggests that combination strategies with agents of complementary mechanisms may allow further dose reduction while preserving or enhancing antitumour efficacy. Notably, the GRAS status of nisin provides a regulatory advantage over synthetic peptide candidates currently in clinical development, potentially shortening the translational timeline if in vivo pharmacokinetic and toxicological profiles are formally established.

### 4.7. Recommendations for Future Research

Future investigations must address five priority areas identified by this review. First, in vivo pharmacokinetic studies comparing free nisin against all major nanocarrier types analyzed here, including PLGA nanoparticles, spectrin-conjugated PLGA, β-cyclodextrin nanosponges, gold nanoparticles, and polymersomes, are urgently needed, as each system presents distinct biodistribution profiles, plasma half-lives, and tumor accumulation kinetics that cannot be inferred from in vitro data alone. Based on the within-line comparative dataset generated here, MDA-MB-231 and HT-29 are the most suitable candidate models for standardized comparative pharmacokinetic studies, having each been evaluated in four independent investigations [25,34,46,71,72].

Second, a head-to-head comparison of nanosystems in the same cell line under identical conditions, particularly PLGA versus β-cyclodextrin formulations, is necessary to identify the most stable and efficacious platform for clinical development. Third, the FZD7/Wnt interaction identified by Balcik-Ercin and Sever 2022 [49] and the p53 reactivation mechanism reported by Mohan Latha Kumari et al. 2025 [45] require experimental validation through genetic knockdown or pharmacological rescue experiments to confirm their causal role in nisin-induced cell death. Fourth, standardization of nisin preparation purity, including mandatory reporting of specific activity in IU/mg and lot-specific HPLC purity data, should be adopted as a minimum reporting requirement across all future studies to enable genuine cross-study comparison [73,74]. Fifth, the synergistic potential of nisin with conventional chemotherapeutics, including curcumin, 5-fluorouracil, and doxorubicin, demonstrated in several included studies, warrants systematic exploration through combination index analysis across multiple cancer lines, with particular attention to triple-negative breast cancer and colorectal cancer models, where the current evidence base is most mature [75,76,77].

## 5. Conclusions

The investigations synthesized in this systematic review provide robust in vitro evidence regarding the antitumor potential of nisin, which is significantly maximized when integrated into advanced pharmaceutical nanosystems. While the bacteriocin alone exhibits cytotoxic activity, this review concludes that its therapeutic viability in oncology is strictly dependent on delivery systems, such as nanoparticles and nanosponges, which enable reductions in IC_50_ values of over 2700×-fold in specific models. However, significant gaps persist, including the shortage of in vivo studies and the lack of experimental standardization regarding nanocarrier characterization. Consequently, future research must prioritize standardized protocols and detailed pharmacokinetic profiling. Only through the integration of rigorous pharmaceutical engineering and complex biological models will it be possible to validate nisin as a viable therapeutic agent in modern oncology.

## Figures and Tables

**Table 1:** Eligibility criteria.

Criterion	Inclusion	Exclusion
Study type	In vitro experimental studies investigating the effect of nisin on tumor cell lines	Observational studies, systematic reviews, in vivo studies, clinical trials, and computational modeling without experimental validation
Population	Cancer cell lines such as melanoma, colon, breast, lung, and glioblastoma	Studies using non-tumor cell models or non-cancerous cell lines
Intervention	Use of advanced nisin delivery systems, such as nanoparticles and nanosponges, or free nisin for comparative baseline purposes	Studies not evaluating nisin or using it in non-oncological contexts, such as food preservation
Comparator	Control cells (untreated) or cells treated with standard drugs such as 5-fluorouracil or doxorubicin	Studies lacking an appropriate control group or providing incomplete/irrelevant results
Outcomes	Reduction in cell viability or induction of apoptosis involving markers such as Bax/Bcl-2 and caspases	Studies evaluating different parameters or providing insufficient data
Language	Studies published in English or Spanish	Studies published in languages other than English or Spanish
Date	No strict temporal restriction, prioritizing studies from the last 6 years	N/A

Abbreviations: N/A = Not applicable; Bax = Bcl-2-associated X protein; Bcl-2 = B-cell lymphoma 2.

**Table 2:** General characteristics of the studied neoplasms.

Neoplasm Type	Cell Line	Relative Impact	Study Reference
Breast	MDA-MB-231, MCF-7	+++	(Haider et al., 2020) [34], (Mohammadi et al., 2024) [42]
	MCF-7	+++	(Salehi et al., 2024) [43], (Khazaei Monfared et al., 2022) [25]
Colon	HT-29, HCT116, SW48, LS180, CaCo-2	+++	(Norouzi et al., 2018) [44], (Mohan Latha Kumari et al., 2025) [45]
	HT-29, SW-620	++	(Haider et al., 2022) [46], (Khazaei Monfared et al., 2022) [25]
Lung	A549, H1299	++	(Saravanakumar et al., 2024) [35], (Patil & Kunda, 2022) [47]
Glioblastoma	SF-767	++	(Ahsan et al., 2024) [48]
Hepatocarcinoma	SNU182, HuH-7	+/−	(Balcik-Ercin & Sever, 2022) [49]
Melanoma	A375	+	(Lewies et al., 2018) [27]
Prostate/Pancreas	PC-3, MiaPaca-2	++	(Haider et al., 2022) [46]

Abbreviations: A375: Human amelanotic melanoma cell line; A549: Human alveolar basal epithelial adenocarcinoma cell line; CaCo-2: Human colorectal adenocarcinoma cell line; H1299: Human non-small cell lung carcinoma cell line; HCT116: Human colorectal tumor cell line; HT-29: Human colorectal adenocarcinoma cell line; HuH-7: Human hepatoma cell line; LS180: Human colorectal adenocarcinoma cell line; MCF-7: Michigan Cancer Foundation-7 breast cancer cell line; MDA-MB-231: M.D. Anderson-Metastatic Breast-231 cell line; MiaPaca-2: Human pancreatic carcinoma cell line; PC-3: Human prostate cancer cell line; SF-767: Human glioblastoma cell line; SNU182: Seoul National University-182 hepatocellular carcinoma cell line; SW48: Human colorectal adenocarcinoma cell line; SW-620: Human colorectal adenocarcinoma cell line. Note: Relative impact is a qualitative assessment based on reported IC_50_ values and overall efficacy. +++ indicates significant inhibition at low doses, ++ indicates moderate inhibition, + indicates minimal inhibition or requirement of very high doses, and +/− indicates qualitative effects where precise IC_50_ values were not reported.

**Table 3:** Intervention details and controls.

Reference	Formulation	Concentrations	Duration	Controls
Khazaei Monfared, Y. et al. 2022 [25]	Nisin-Z in β-CD-NS (CDI/PMDA)	62–250 µg/mL	24 h	NC: Empty NS; PC: Oxaliplatin, Triton-X
Ahsan, H. et al. 2024 [48]	Free nisin (bioinformatics + MTT validation)	1–100 µg/mL	48 h	NC: CHO cells, PBS; PC: N/A
Balcik-Ercin, P. et al. 2022 [49]	Free nisin	5–320 µg/mL	48 h	NC: Culture medium; PC: N/A
Haider, T. et al. 2020 [34]	Nisin in PLGA NPs; SPN-conjugated NPs	0.05–500 µg/mL	48 h	NC: FR-2 cells; PC: N/A
Lewies et al. 2018 [27]	Nisin-Z (95% purity)	50–400 µM	24 h	NC: Vehicle, HaCaT; PC: Triton-X, FCCP
Saravamakumar et al. 2024 [35]	Nisin-cyst-PE-GNPs (GSH-responsive)	1–100 µg/mL	48 h	NC: NIH3T3 cells; PC: Triton-X, PBS
Norouzi et al. 2018 [44]	Free nisin	20–450 IU/mL	24 h	NC: Untreated cells; PC: N/A
Mohan Latha Kumari et al. 2025 [45]	Free nisin	6.25–100 µg/mL	24 h	NC: Untreated cells; PC: N/A
Patil and Kunda, 2022 [47]	Free nisin-ZP	0–250 µM	48 h	NC: Untreated cells, fR2; PC: N/A
Haider, T. et al., 2022 [46]	Nisin-PLGA NPs (NPN)	5–250 µg/mL	48 h	NC: FR-2 cells; PC: 5-FU, Doxorubicin, Paclitaxel
Mohammadi et al., 2024 [42]	Glycated nisin A	5–80 µg/mL	24–72 h	NC: DMSO; PC: N/A
Salehi et al., 2024 [43]	Nisin + Curcumin in polymersomes	5–40 µg/mL	24–48 h	NC: HDF cells; PC: N/A

Abbreviations: βCD-NS: β-cyclodextrin nanosponges; CDI: Carbonyldiimidazole; PMDA: Pyromellitic dianhydride; PLGA NPs: Poly(lactic-co-glycolic acid) nanoparticles; NPs: Nanoparticles; FR-2: Normal mammary epithelium; CUR: Curcumin; NC: Negative control; PC: Positive control; OxPt: Oxaliplatin; CHO: Chinese hamster ovary cells; PBS: Phosphate-buffered saline; N/A: Not applicable; HDF: Human dermal fibroblasts; HaCaT: Non-malignant keratinocytes; FCCP: Carbonyl cyanide 4-(trifluoromethoxy)phenylhydrazone; Nisin-cyst-PE-GNPs: Gold nanoparticles functionalized with nisin and modified with cysteamine. Note on units: Due to the variability in reporting within the primary studies, concentrations are presented in μM, μg/mL, or IU/mL according to the original source.

**Table 4:** Techniques used to quantify cell viability and apoptosis.

Study Reference	Cell Viability	Apoptosis	Statistical Methods
Khazaei Monfared, Y. et al. 2022 [25]	MTT, LDH	Annexin V/PI	GraphPad Prism 8, ANOVA
Ahsan, H. et al. 2024 [48]	MTT	N/A	GraphPad Prism 9, Student’s *t*-test
Balcik-Ercin, P. et al. 2022 [49]	N/A	BD Accuri C6	Student’s *t*-test, ANOVA
Haider, T. et al. 2020 [34]	SRB	N/A	GraphPad Prism 8, ANOVA
Lewies et al. 2018 [27]	MTT, LDH, NR, TR	Annexin V/PI	GraphPad Prism 5, ANOVA
Saravamakumar et al. 2024 [35]	WST	AO/EB	OriginPro 8.5, ANOVA
Norouzi et al. 2018 [44]	MTT, TR	N/A	Student’s *t*-test
Mohan Latha Kumari et al. 2025 [45]	Alamar Blue	AO/EB, ELISA	ANOVA with Dunnett’s test
Patil and Kunda, 2022 [47]	MTT	Annexin V/PI	GraphPad Prism 9, ANOVA
Haider, T. et al., 2022 [46]	SRB	DAPI staining	GraphPad Prism 8, ANOVA
Mohammadi et al., 2024 [42]	MTT, TR	Annexin V/PI, AO/EB	GraphPad Prism 10, ANOVA
Salehi et al., 2024 [43]	MTT	Annexin V/PI	GraphPad Prism 8, ANOVA

Abbreviations: MTT = 3-(4,5-dimethylthiazol-2-yl)-2,5-diphenyltetrazolium bromide; LDH = Lactate Dehydrogenase; SRB = Sulforhodamine B; NR = Neutral Red; TR = Trypan Blue; PI = Propidium iodide; WST = Water-Soluble Tetrazolium; AO/EB = Acridine Orange/Ethidium Bromide; DAPI = 4′,6-diamidino-2-phenylindole; N/A = Not applicable.

**Table 5:** Comparative analysis of nisin efficacy across cancer cell line models, grouped by cell line.

Cell Line	Cancer Type	Formulation	IC_50_ Free Nisin	IC_50_ Nanosystem	Fold ↓	Key Mechanism/Findings	Ref.
MDA-MB-231—Triple-Negative Breast Cancer							
MDA-MB-231	Breast (TNBC)	PLGA-SPN NPs	162.38 µg/mL	0.06 µg/mL	>2706×	PS/PE membrane targeting; Bcl-2 ↓; Bak/Bax ↑; DΨm ↓; SI > 2383× vs. FR-2	Haider et al., 2020 [34]
MDA-MB-231	Breast (TNBC)	Glycated nisin	11.64 µg/mL (72 h)	0.05 µg/mL (72 h)	~233×	Apoptosis 73–81% at 24 h; negligible necrosis; colony inhibition	Mohammadi et al., 2024 [42]
MDA-MB-231	Breast (TNBC)	PLGA NPs (NPN)	162 µg/mL	13.0 µg/mL	12.5×	ROS ↑; DΨm ↓; Bcl-2 ↓ (Western blot); DAPI nuclear fragmentation	Haider et al., 2022 [46]
MDA-MB-231	Breast (TNBC)	GNPs (nisin-cyst-PE)	NR	>100 µg/mL	N/A	GSH-responsive; lower sensitivity than A549; ROS ↑	Saravanakumar et al., 2024 [35]
MCF-7—Breast Cancer (Luminal)							
MCF-7	Breast	Polymersomes (Ni + CUR)	43.56 µg/mL (24 h) LD_50_ †	18.20 µg/mL (24 h) LD_50_ †	2.4×	Synergistic effect (CI < 1); apoptosis 40.9%; endocytosis ↑; HDF as normal control	Salehi et al., 2024 [43]
MCF-7	Breast	β-CD nanosponges (PMDA)	NR ‡	~30% viability at 250 µg/mL	N/A	Early apoptosis; lower uptake than HT-29 (FACS, *p* < 0.0001)	Khazaei Monfared et al., 2022 [25]
MCF-7	Breast	PLGA NPs (NPN)	NR	46.13 µg/mL	—	Lower sensitivity than MDA-MB-231; dose-dependent response	Haider et al., 2022 [46]
HT-29—Colorectal Adenocarcinoma							
HT-29	Colorectal	Free nisin	350–800 IU/mL §	—	—	CEA ↓; CEAM6 ↓; MMP2F ↓; MMP9F ↓ (qRT-PCR + ELISA); anti-metastatic	Norouzi et al., 2018 [44]
HT-29	Colorectal	Free nisin	68.09 µg/mL	—	—	p53 ↑; caspases 3/7/9 ↑; G0/G1 arrest (52.4% → 72.1%); first p53 reactivation report	Mohan Latha Kumari et al., 2025 [45]
HT-29	Colorectal	β-CD nanosponges (PMDA)	NR ‡	~20% viability at 250 µg/mL	N/A	LDH ↑↑; late apoptosis; PMDA-NS > CDI-NS; uptake > MCF-7	Khazaei Monfared et al., 2022 [25]
HT-29	Colorectal	PLGA NPs (NPN)	—	180 ± 1.43 µg/mL	—	Sustained release; moderate cytotoxic response	Haider et al., 2022 [46]
A549/H1299—Lung Cancer							
A549	Lung	GNPs (nisin-cyst-PE)	62.5 µg/mL	0.88 µg/mL	71×	GSH-triggered release; ROS ↑; AO/EB apoptosis; A549 >> MDA-MB-231 selectivity	Saravanakumar et al., 2024 [35]
A549	Lung	Free nisin-ZP	444.3 µg/mL	—	—	G0/G1 arrest; ROS ↑; DΨm ↓; 3D spheroid inhibition at 250 µM; p53-independent	Patil & Kunda, 2022 [47]
H1299	Lung	Free nisin-ZP	460.5 µg/mL	—	—	p53-null line; similar response to A549; p53-independence confirmed	Patil & Kunda, 2022 [47]
Other Cancer Lines							
SF-767	Glioblastoma	Free nisin (+docking)	30.65 µg/mL	—	—	CHO IC_50_ = 110.4 µg/mL; SI = 3.6×; GCSF/JAK-STAT pathway docking	Ahsan et al., 2024 [48]
HuH-7	HCC	Free nisin	IC_50_ NR; 83.3% inh. at 160 µg/mL	—	—	G2/M + S phase arrest; TWIST1 ↓ (EMT); FZD7 docking (−6.23 kcal/mol)	Balcik-Ercin & Sever, 2022 [49]
SNU182	HCC	Free nisin	IC_50_ NR; 78.4% inh. at 160 µg/mL	—	—	Apoptosis 37.5% at 320 µg/mL; TWIST1 ↓; mesenchymal phenotype	Balcik-Ercin & Sever, 2022 [49]
A375	Melanoma	Free nisin-Z	188.5 ± 8.7 µM	—	—	SI = 2.3× vs. HaCaT (439 ± 8.3 µM); bioenergetic collapse; ROS ↑; invasion ↓	Lewies et al., 2018 [27]

Abbreviations: IC50: Half-maximal inhibitory concentration; LD50: Lethal dose 50%; NR: Not reported; N/A: Not applicable; PLGA: Poly(lactic-co-glycolic acid); NPs: Nanoparticles; NPN: Nisin-loaded PLGA nanoparticles; SPN: Substance P-conjugated nanoparticles; GNPs: Gold nanoparticles; β-CD: β-cyclodextrin; NS: Nanosponges; CDI: Carbonyldiimidazole; PMDA: Pyromellitic dianhydride; GSH: Glutathione; CUR: Curcumin; CI: Combination index; PS: Phosphatidylserine; PE: Phosphatidylethanolamine; ROS: Reactive oxygen species; DΨm: Mitochondrial membrane potential; LDH: Lactate dehydrogenase; CEA: Carcinoembryonic antigen; CEAM6: CEA-related cell adhesion molecule 6; MMP2F/MMP9F: Matrix metalloproteinase 2F/9F; EMT: Epithelial–mesenchymal transition; SI: Selectivity index; TNBC: Triple-negative breast cancer; HCC: Hepatocellular carcinoma; Bcl-2: B-cell lymphoma 2 (anti-apoptotic protein); Bak: Bcl-2 homologous antagonist/killer; Bax: Bcl-2-associated X protein; p53: Tumor protein p53; TWIST1: Twist-related protein 1 (EMT transcription factor); FZD7: Frizzled-7 re-ceptor; GCSF: Granulocyte colony-stimulating factor; JAK-STAT: Janus kinase–signal transducer and activator of transcription pathway; Nisin-ZP: Nisin Z peptide; FR-2: Normal mammary epithelium cell line; CHO: Chinese hamster ovary cell line; HDF: Human dermal fibroblasts; HaCaT: Non-malignant human keratinocyte cell line; DAPI: 4′,6-diamidino-2-phenylindole (nuclear stain); AO/EB: Acridine orange/ethidium bromide (dual fluorescence staining); FACS: Fluorescence-activated cell sorting; qRT-PCR: Quantitative reverse transcription polymerase chain reaction; ELISA: Enzyme-linked immunosorbent assay; G0/G1: Gap 0/Gap 1 phases of the cell cycle; G2/M: Gap 2/Mitosis phases of the cell cycle; IU/mL: International Units per milliliter. Symbols: ↑: increased or upregulated; ↓: decreased or downregulated; ↑↑: markedly increased; Fold ↓: fold decrease in IC50 relative to free nisin; →: transi-tion or change (e.g., cell cycle shift); >>: substantially greater than; —: data not available or not applicable for that column. † Values reported as LD50 instead of IC50. ‡ Precise IC50 values were not reported; cytotoxicity is ex-pressed as percentage of viability at a given concentration. § Concentrations originally reported in IU/mL rather than µg/mL or µM.

## Data Availability

No new data were created or analyzed in this study. Data sharing is not applicable to this article.

## Supplementary Materials

The following supporting information can be downloaded at: https://www.mdpi.com/article/10.3390/ph19040611/s1, Table S1: PRISMA Checklist. Reference [78] is cited in the supplementary materials.

## Abbreviations

The following abbreviations are used in this manuscript:

**Abbreviation**	**Definition**
PRISMA	Preferred Reporting Items for Systematic Reviews and Meta-Analyses
PICO	Population, Intervention, Comparison, and Outcome
PROSPERO	International Prospective Register of Systematic Reviews
MeSH	Medical Subject Headings
FDA	Food and Drug Administration
GRAS	Generally Recognized as Safe
TNBC	Triple-Negative Breast Cancer
HCC	Hepatocellular Carcinoma
EMT	Epithelial–Mesenchymal Transition
MOMP	Mitochondrial Outer Membrane Permeabilization
GSH	Glutathione
ROS	Reactive Oxygen Species
ΔΨm	Mitochondrial Membrane Potential
PS	Phosphatidylserine
PE	Phosphatidylethanolamine
SI	Selectivity Index
FZD7	Frizzled-7 receptor
βCD-NS	β-Cyclodextrin nanosponges
CDI	Carbonyldiimidazole
PMDA	Pyromellitic dianhydride
PLGA NPs	Poly(lactic-co-glycolic acid) nanoparticles
SPN	Spectrin-conjugated nanoparticles
GNPs	Gold nanoparticles
Nisin-cyst-PE-GNPs	Gold nanoparticles functionalized with nisin and modified with cysteamine
CUR	Curcumin
NC	Negative control
PC	Positive control
OxPt	Oxaliplatin
CHO	Chinese hamster ovary cells
HDF	Human dermal fibroblasts
HaCaT	Non-malignant keratinocytes
PBS	Phosphate-buffered saline
FCCP	Carbonyl cyanide 4-(trifluoromethoxy)phenylhydrazone
N/A	Not applicable
MTT	3-(4,5-dimethylthiazol-2-yl)-2,5-diphenyltetrazolium bromide
LDH	Lactate dehydrogenase
SRB	Sulforhodamine B
NR	Neutral red
TR	Trypan blue
PI	Propidium iodide
WST	Water-soluble tetrazolium
AO/EB	Acridine Orange/Ethidium Bromide
DAPI	4′,6-diamidino-2-phenylindole
IC_50_	50% inhibitory concentration
LD_50_	50% lethal dose
CEA	Carcinoembryonic antigen
CEAM6	Carcinoembryonic antigen-related cell adhesion molecule 6
MMP	Matrix metalloproteinase
MMP2F	Matrix metalloproteinase 2F
MMP9F	Matrix metalloproteinase 9F
TWIST1	Twist family BHLH transcription factor 1
